# FnCas12a/crRNA-Mediated Genome Editing in *Eimeria tenella*

**DOI:** 10.3389/fgene.2021.738746

**Published:** 2021-09-22

**Authors:** Peipei Cheng, Zhihao Zhang, Fayu Yang, Shuo Cai, Lina Wang, Chunmei Wang, Mi Wang, Yingchun Liu, Chenzhong Fei, Lifang Zhang, Feiqun Xue, Feng Gu

**Affiliations:** Key Laboratory of Veterinary Chemical Drugs and Pharmaceutics, Ministry of Agriculture and Rural Affairs, Shanghai Veterinary Research Institute, Chinese Academy of Agricultural Sciences, Shanghai, China

**Keywords:** *Eimeria tenella*, FnCas12a, genome editing, knock-in, *EtHistone H4*, *EtActin*

## Abstract

*Eimeria* species are intracellular parasites residing inside the intestinal epithelial cell, which cause poultry coccidiosis and result in significant financial losses in the poultry industry. Genome editing of *Eimeria* is of immense importance for the development of vaccines and drugs. CRISPR/Cas9 has been utilized for manipulating the genome of *Eimeria tenella* (*E. tenella*). Ectopic expression of Cas9, i.e., *via* plasmids, would introduce transgene, which substantially limits its application, especially for vaccine development. In this study, we initially optimized the condition of the transfection protocol. We demonstrated that with the optimized condition, the transfection of FnCas12a (also known as “FnCpf1”) protein and crRNA targeting *EtHistone H4* triggered DNA double-strand breaks *in vivo*. We then used this strategy to knock-in a coding cassette for an enhanced yellow fluorescent protein (*EYFP*) and dihydrofolate reductase–thymidylate synthase gene (*DHFR*) as a selection marker to tag endogenous *EtActin*. The engineered *E. tenella* parasite possesses *EYFP* expression in its entire life cycle. Our results demonstrated that FnCas12a could trigger genome editing in *E. tenella*, which augments the applicability of the dissection of gene function and the development of anticoccidial drugs and vaccines for *Eimeria* species.

## Introduction

Coccidiosis is one of the most important animal parasitic diseases. It is reported in several species, including chickens, ducks, puppies, rabbits, piglets, dogs, horses, kittens, and birds, and is caused by the members of the *Eimeria* genus (Stock et al., 2018). Chicken coccidiosis is an intestinal disease caused by one or more single-cellular apicomplexan protozoa and characterized by diarrhea, blood stool, reduced feed conversion rate, growth retardation, and death (Ding et al., 2012; Sun et al., 2014). It causes a significant impact on the poultry industry, where the high-density housing of susceptible birds is favorable to parasite transmission (Berezin et al., 2010; Blake and Tomley, 2014; Blake et al., 2020). *Eimeria tenella* (*E. tenella*) is one of the highly pathogenic *Eimeria* species (Lopez-Osorio et al., 2020).

The genome of avian species *E. tenella* comprises approximately 60 Mbp DNA (Shirley, 2000), with approximately 8,618 genes (Cai et al., 2003). Over the past decades, several techniques such as immunofluorescence localization, recombinant proteins, Western blotting, RT-PCR, and protein pull-down assay have been used to delineate the function of genes for cell cycle (Diallo et al., 2019), invasion (Li et al., 2020a,b), and drug resistance (Yu et al., 2021). However, due to the lack of genome manipulation tools, the function of only a very few genes has been well deciphered (Blake, 2015).

In recent years, CRISPR/Cas [clustered regularly interspaced short palindromic repeats (CRISPR)/associated endonuclease (Cas)] has been adopted as a tool to manipulate the genomes of cultured cells, animals, and plants, thereby accelerating the pace of fundamental research and enabling clinical and agricultural breakthroughs (Cong et al., 2013; Hwang et al., 2013; Jiang et al., 2013; Shan et al., 2013; Wang et al., 2013). Specifically, a prokaryotic RNA programmable nuclease system can introduce a double-strand break (DSB) at the target site on the genome through the expression of Cas9 nuclease and a targeting single-guide RNA (sgRNA; Jinek et al., 2012). Then, the DSBs are repaired either by the error-prone nonhomologous end-joining pathway (NHEJ; Bibikova et al., 2002), generating deletions or insertions, or by homology-directed repair (HDR) if a donor DNA template is present (Urnov et al., 2005). CRISPR/Cas has been successfully applied in several protozoan parasites, including *Plasmodium* (Zhang et al., 2014; Vanaerschot et al., 2017), *Toxoplasma* (Sidik et al., 2018; Young et al., 2019), *Cryptosporidium* (Vinayak et al., 2015), *Leishmania* (Zhang and Matlashewski, 2015; Baker et al., 2021), and *Trypanosoma* (Peng et al., 2014; Soares Medeiros et al., 2017).

Regarding the genome manipulation of *E. tenella*, transient transfection was first reported by Kellerher and Tomely in 1998 (Kelleher and Tomley, 1998), before stable transgenesis was described by Clark in 2008 (Clark et al., 2008). Several studies have been performed for optimization of the transgenic promoters, fluorescent reporter genes, fusion strategy, and selection for enrichment of positive transformants (Clark et al., 2008; Hanig et al., 2012; Tang et al., 2016; Pastor-Fernandez et al., 2019), i.e., using the selection marker dihydrofolate reductase–thymidylate synthase (*DHFR*) gene (Liu et al., 2008). Due to the lack of a method providing a continuous culture of *Eimeria* at one stage as *Toxoplasma*, no genome editing techniques have been reported until the past year (Hu et al., 2020; Tang et al., 2020). It was reported that an *E. tenella* parasite strain harboring eCas9 expression would facilitate gene manipulation. Using this specific strain, the plasmid for sgRNA expression plus the donor fragment would trigger genome editing (Hu et al., 2020). The other study used the CRISPR/Cas9 plasmid system successfully tagged the endogenous microneme protein 2 (*EtMic2*) with a red fluorescent protein (Tang et al., 2020). These advances promoted the functional genomics study of *Eimeria* species.

*Streptococcus pyogenes* Cas9 (SpCas9) is one of the most commonly used Cas9 variants for genome editing. In addition, Cas12a (also known as “Cpf1”), a class 2 CRISPR/Cas family of nucleases, was reported to be programmable and highly specific, with efficiencies being comparable to those of SpCas9 nuclease (Zetsche et al., 2015). To date, at least three Cas12a proteins from *Lachnospiraceae bacterium* (LbCas12a), *Acidaminococcus* sp. (AsCas12a), and *Francisella novicida U112* (FnCas12a) have been adopted as genome editing tools, respectively (Tu et al., 2017). Cas12a recognizes a thymine-rich protospacer adjacent motif (PAM) sequence at the 5' end of the protospacer, which significantly increases the range of CRISPR-endonuclease-editable genomic sites. It creates a staggered DSB that results in 5' overhangs distal to the PAM site, compared with Cas9 that generates blunt-end cleavage products proximal to the PAM site (Tu et al., 2017; Lin et al., 2018). In particular, Cas12a has low off-target effects (Kim et al., 2016). CRISPR-Cas12a requires only a single crRNA without a tracrRNA, which is composed of a 19-nt (nucleotide) direct repeat (DR), followed by a 21- to 25-nt spacer sequence (i.e., FnCas12a with a 21-nt spacer sequence; Tu et al., 2017). Compared with AsCas12a and LbCas12a, FnCas12a recognizes a 5'-KYTV-3' PAM in human cells, which provides more target sequences for editing. Recently, Cas12a has been successfully applied in malarial parasites (Nessel et al., 2020; Zhao et al., 2020).

In this study, using FnCas12a–RNP (FnCas12a protein and crRNA), we developed a protocol to manipulate the genome of *E. tenella*. Specifically, we optimized the transfection programs of *E. tenella* sporozoites. Then, *EtHistone H4* gene in *E. tenella* parasite was successfully disrupted using FnCas12a–RNP. Moreover, a fluorescent protein-encoding gene (*EYFP*) was tagged to *EtActin* gene in the presence of FnCas12a–RNP and donor DNA template. Our results demonstrated that *EYFP* was constitutively expressed throughout the life cycle of *E. tenella*. These results demonstrated that FnCas12a–RNP could be used for manipulating the *E. tenella* genome. This technique would significantly augment the development of vaccines and drugs against *Eimeria* species as well as the dissection of novel gene functions.

## Materials and Methods

### Animals and Parasites

This study was approved by the Ethics Committee of our institute. One-day-old Pudong yellow chickens were purchased from a local hatchery (Min You, Shanghai, China). The wild-type *E. tenella* strain used was maintained in our laboratory. The collection, sporulation, and purification of coccidia procedures were conducted as described previously (Dulski and Turner, 1988; Pastor-Fernandez et al., 2019).

### Dosage of Pyrimethamine Used for Screening *E. tenella*

A total of 144 five-day-old chickens were divided into six groups. Each group consisted of 24 chickens and was further subdivided into three cages, with eight chickens in one cage. *E. tenella* sporozoites (1×10^7^) were inoculated into 24 chickens through the cloaca (approximately 5×10^4^ oocysts/chicken). The first group was fed without pyrimethamine. From the second to sixth groups, the feed was supplemented with pyrimethamine at dosages of 100, 150, 200, 250, and 300mg/kg, respectively. Fecal oocysts were measured from 5 to 11days post-infection (d.p.i.). The total number of oocysts was calculated using the modified McMaster chamber method (Haug et al., 2006).

### Plasmid Construction

To measure the transfection efficiency using the Lonza 4D-Nucleofector, we generated an expression cassette to determine the transfection efficiency. The pyrimethamine-resistant gene TgDHFR-ts-m2m3 (*DHFR*) and the *EYFP* gene encoding the enhanced yellow fluorescent protein were driven by a *EtHistone H4* promoter (1,000bp). The primers used for amplifying the fragment are listed in Supplementary Table S1.

To generate the *EtActin* gene template for homologous recombination, we first amplified the C-terminal part of the coding region (742bp) as the left arm and 751bp from the 3′UTR region following of the stop codon as the right homologous arm of the *EtActin* gene by PCR using the primers listed in Supplementary Table S1. A DNA fragment encoding p2A-DHFR-EYFP was inserted between the left and right arms in frame of *EtActin* gene. The homologous recombination templates have SnaBI restriction sites flanking the left and right homologous arms. Plasmid DNA and genomic DNA were isolated by standard techniques. The desired specific sequence in the constructs was confirmed by DNA sequencing.

### Expression of FnCas12a Protein and DNA Cleavage by FnCas12a–RNP *in vitro*

The purification method of FnCas12a and the synthesis of crRNA *in vitro* were performed as described by Wang et al. (2019). The coding sequence of FnCas12a is obtained from plasmid #69976 (Addgene), and the plasmid sequence for the expression of FnCas12a in *E. coli* is provided in Supplementary Material. The FnCas12a protein was purified using the BeyoGold™ His-tag Purification Resin (P2210, Beyotime Biotechnology) according to the manufacturer’s instructions. The FnCas12a protein was eluted with 200mM imidazole elution buffer (200mM imidazole, 50mM NaH_2_PO_4_, 300mM NaCl, pH 8.0; the detailed protocol is listed in Supplementary Protocol). The concentration of FnCas12a was measured using BCA Protein Assay Kit (Beyotime Biotechnology, China). The purified FnCas12a proteins were analyzed by sodium dodecyl sulfate–polyacrylamide gel electrophoresis (SDS-PAGE) and coomassie blue staining. The MEGAscript T7 Transcription Kit (Thermo Fisher Scientific, Waltham, MA, USA) was used to synthesize crRNA *in vitro* (Supplementary Table S2).

Regarding the *in vitro* cleavage, we used PCR fragment with the template of *EtHistone H4* and its crRNA-1 to optimize the ratio of RNP/template (0:1, 2:1, 4:1, 10:1, 20:1, 30:1, and 40:1) at 37°C with the reaction time of 60min, the reaction time (10, 20, 30, 60, 90, and 120min) with the molar ratio of 30:1 (RNP/template) at 37°C, and the temperature (16, 28, 37, 41, 44, 50, 60, 70, and 80°C) with the molar ratio of 30:1 (RNP/template) and time of 10min. In this reaction, 190ng of *EtHistone H4* PCR fragment (575bp) harboring the target sites for genome editing was mixed with the purified FnCas12a/crRNA (molar ratio 1:1; RNP) complex in a reaction buffer (10mM Tris-HCl, 10mM MgCl_2_, 100mM NaCl, and 1mM DTT, pH 7.4) and incubated at the different temperatures and times. To determine the activity of three crRNAs for *EtHistone H4* gene, we set the molar ratio of RNP/template at 10:1 and the reaction at 37°C for 60min. Here, 200ng of PCR products from the templates of *EtActin* was used. Finally, agarose gel electrophoresis was used to detect the effect of cleavage. The DNA cleavage efficiency was calculated using the ImageJ software (version 1.47) as described previously (Wang et al., 2019).

### Optimization of the Transfection Programs

Initially, 22 transfection programs (EH100, EO100, DN100, ER100, FA100, CM150, FP167, EZ158, ES100, FI158, FF158, FF190, FF191, FI189, FI190, FI115, FI191, FI169, FL158, FL190, FL191, and EO114) were assessed using the Lonza 4D-Nucleofector with small transfection volume (20μl). Then, additional studies with larger transfection volume (100μl) were performed to compare the transfection efficiency of selected programs *in vivo* and *in vitro*. The detailed protocol is provided in Supplementary Material.

### Parasite Transfection

*E. tenella* sporozoites were purified from freshly purified oocysts by bile-trypsin digestion and DE-52 cellulose filtration (Pastor-Fernandez et al., 2019). *E. tenella* sporozoites (1×10^7^) were transfected with 30μg of FnCas12a–RNP and 40μg of linearized plasmid (only for homologous recombination) in a 100-μl Nucleocuvette using the Lonza 4D-Nucleofector with the program EH100 and incubated at 41°C for 15min. The transfected parasites were inoculated into 3-day-old chickens through the cloaca and selected using 150mg/kg pyrimethamine (Pyr) in feed 18h after inoculation. Oocysts were collected from feces at 6–10days after inoculation. The edited parasites were enriched by feeding with approximately 250 and 300mg/kg of pyrimethamine in the second and third passages, respectively.

### Primary Chicken Kidney Cell and MDBK Cell Cultural and Parasite Infection *in vitro*

Primary chicken kidney (PCK) cells were isolated from 3-day-old chickens and cultured as described previously (Tang et al., 2016). After the addition of sporozoites, 5% FBS was used to incubate PCK cells. Madin–Darby bovine kidney (MDBK) cells were cultured in the same manner as done for PCK cells. Extracellular parasites were removed at 24h after the addition of sporozoites in cells and washed three times with phosphate-buffered saline, after which a new medium was added to continue culture (Thabet et al., 2017). The growth of the parasites was observed under a fluorescence microscope.

### Targeted Deep Sequencing and Data Analysis

The oocysts were purified using sodium hypochlorite. Targeted deep sequencing was performed as described previously (Wang et al., 2019). Briefly, fragments of *EtHistone H4* harboring target site were amplified. Barcodes and sequencing indexes was used to construct the library for deep sequencing (NovaSeq 6,000, Illumina, San Diego, CA, United States). Trimmomatic (version 0.36) was utilized to remove adaptors and low-quality reads from the resulting 150bp paired-end reads. The software Bowtie 2 (version 2.3.3) was used to map the reads to the template. Indels were identified by CRISPResso (version 1.0.8) with high-quality reads (above Q30). The results of NGS data are available at the NCBI Sequence Read Archive under the Bioproject ID PRJNA731675 (sample accession numbers SAMN19296119 and SAMN19296120). Potential off-target sites were predicted by Cas-OFFinder^1^ with maximum three nucleotides mismatch. The *E. tenella* reference genome from NCBI was downloaded and used for prediction of the potential off-target sites. For the genome editing of *EtActin*, targeted modification was confirmed by PCRs using two pairs of primer to confirm the knock-in. The sequences of PCR products were obtained by inserting them into the vector pJET1.2 and then subjecting them to Sanger sequencing.

### Statistical Analysis

GraphPad Prism 8.0 was used to generate graphs. All data were expressed as mean±SEM. Differences were determined by two-tailed Student’s *t* test between two groups or one-way ANOVA followed by post hoc Bonferroni test for group comparison. The software of SPSS statistics 22.0 (SPSS Inc., Chicago, IL, USA) was used for statistical analysis. The criterion for statistical significance was ^*^p<0.05, ^**^p<0.01, and ^***^p<0.001.

## Results

### DNA Cleavage Activity by FnCas12a–RNP *in vitro*

**Figure 1:**
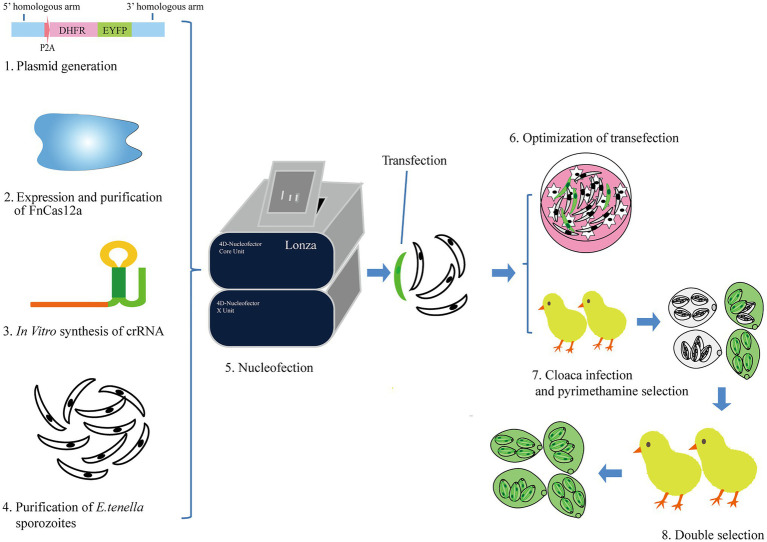
The diagram for genetic manipulation of FnCas12a/crRNA-mediated genome editing in *E. tenella*. (1) The generation of plasmids used as homologous recombination template. (2) Expression and purification of FnCas12a proteins. (3) crRNA synthesis *in vitro*. (4) The purification of *E. tenella* sporozoites. (5) *E. tenella* sporozoites were transfected with FnCas12a protein, crRNA with or without homologous recombination template using Lonza 4D-Nucleofector. (6) Sporozoites (2×10^6^) were cultured in PCK cells to show transfection results. We also used this step to compare the transfection efficiency of different nucleofector programs. (7) The remaining part of sporozoites (8×10^6^) was inoculated in chicken *via* cloaca and selected with pyrimethamine in feed. The progeny oocysts were harvested from feces 6–10days after inoculation. (8) The progeny oocysts infected new batches of chickens (coccidia-free) by oral gavage. Pyrimethamine was used to enrich for parasites carrying the DHFR gene.

Our previous study demonstrated that using SaCas9-RNP we can manipulate the human genome (Wang et al., 2019). However, SaCas9 requires a 6-nt PAM for target site recognition, thus limiting its use for target selection. In the present study, we evaluated whether FnCas12a could be utilized for the manipulation of the *E. tenella* genome. We designed the genome editing protocol, which included RNP preparation, nucleofection (or electroporation), and *in vivo* maturation of oocysts (Figure 1). Initially, we induced the expression of the recombinant FnCas12a protein in *Escherichia coli*. The results of SDS-PAGE showed that FnCas12a was successfully expressed (Supplementary Figure S1). His-tagged FnCas12a was purified from the supernatants of cell lysates (Supplementary Figure S1). We then tested the function of the recombinant FnCas12a protein *in vitro*.

**Figure 2:**
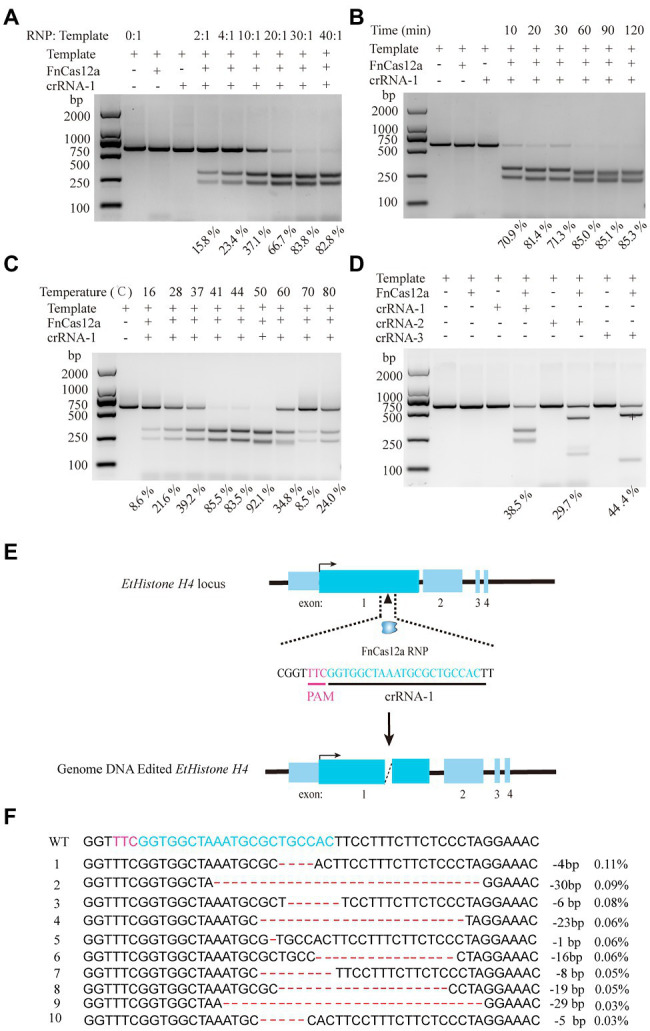
FnCas12a-mediated gene knock-out in *E. tenella*. **(A)** The effect of the molar ratio of RNP/template on *in vitro* cleavage efficiency of the *EtHistone H4* fragment. The molar ratio of RNP/template at 0:1, 2:1, 4:1, 10:1, 20:1, 30:1, 40:1, respectively, with the temperature at 37°C and reaction time of 60min. **(B)** The effects of incubation times. The reaction time of 10, 20, 30, 60, 90, and 120min with the temperature at 37°C and the molar ratio of RNP/template at 30:1. **(C)** The effects of reaction temperature for the cleavage. The reaction at the temperature of 16, 28, 37, 41, 44, 50, 60, 70, and 80°C, respectively, with the molar ratio of RNP/template was 30:1 and reaction time of 10min. **(D)** Comparison of cleavage activity of three crRNAs. The molar ratio of RNP/template at 10:1, incubated in 37°C for 60min. The edited products were quantified with agarose gel electrophoresis. **(E)** Schematic diagram of FnCas12a/crRNA-1 trigger genome editing in *EtHistone H4*. **(F)** The top 10 indels were obtained *via* next-generation sequencing.

Because *Histone H4* is one of the five major histone proteins involved in the structure of chromatin in eukaryotic cells, which is a core component of the nucleosome and plays a vital role in DNA repair, DNA replication, transcription regulation, and chromosomal stability (Grover et al., 2018; Kumar et al., 2020), we selected *EtHistone H4* as the target gene. The fragment of *EtHistone H4* harboring the target sites for cleavage was amplified and then inserted into the pJET1.2 vector. Cleavage was performed using FnCas12a–RNP *in vitro*. We also tested different conditions to optimize cleavage. Results showed that the recombinant FnCas12a protein plus crRNA (*EtHistone H4*) can trigger cleavage. The optimal ratio of RNP/template was 30:1 (molar ratio) at the temperature of 37°C (Figure 2A). Sixty minutes is satiated for FnCas12a-RNP cleavage activity *in vitro* with the molar ratio of RNP/template of 30:1 at 37°C (Figure 2B). Surprisingly, at 41–50°C, FnCas12a exhibited much higher activity in the reaction time of 10min, and the cutting efficiency was 92% (Figure 2C; Supplementary Figure S2). We also evaluated two additional crRNAs with the molar ratio of RNP/template at 30:1 and found that all these crRNAs exhibited similar activities. Hence, we selected the molar ratio of RNP/template at 10:1 to perform a further study of *in vitro* cleavage activity, which resulted in crRNA-1 and crRNA-3 possessing relatively higher activities (Figure 2D; Supplementary Figure S3). Collectively, the above-described results illustrated that with the optimized condition, FnCas12a–RNP can trigger robust DNA cleavage *in vitro*.

### FnCas12a-Mediated Knock-Out

Next, we attempted to knock-out *EtHistone H4 via* FnCas12a-mediated genome editing. Purified *E. tenella* sporozoites (1×10^7^) were transfected with 30μg of FnCas12a/crRNA-1 (1:1) and incubated at 41°C for 15min (Figure 2E). Chickens were inoculated with the transfected sporozoites through the cloaca. We then collected the oocysts from the feces of chickens from 6 to 10days after infection. In addition, the oocysts isolated from caecum, and the PCK cells with transfected sporozoites were also collected. The genomic DNA of purified oocysts and cells were isolated using the phenol chloroform method, and on-target cleavage efficiencies were estimated *via* next-generation sequencing. Thereby, we discovered the indel rates at the *EtHistone H4* target site of oocysts isolated from feces, oocysts isolated from caecum, and the PCK cells with transfected sporozoites were about 1.20, 1.23, and 1.53%, respectively. The indel products included 1- to 30-bp deletions at the target cleavage sites (Figure 2F). No off-target sites were found in *E. tenella* genome with up to three nucleotides mismatch between the crRNA and potential off-target sites. Meanwhile, if there are more than two nucleotides mismatch, FnCas12a can efficiently distinguish and reject the off-target sites (Liu et al., 2021). Thus, we assume a high specificity of gene manipulation at the desired target locus. This result demonstrated that the expected deletion of *EtHistone H4* gene at specific sites could be successfully achieved through FnCas12a-mediated genome editing.

### FnCas12a-Mediated Knock-In

**Figure 3:**
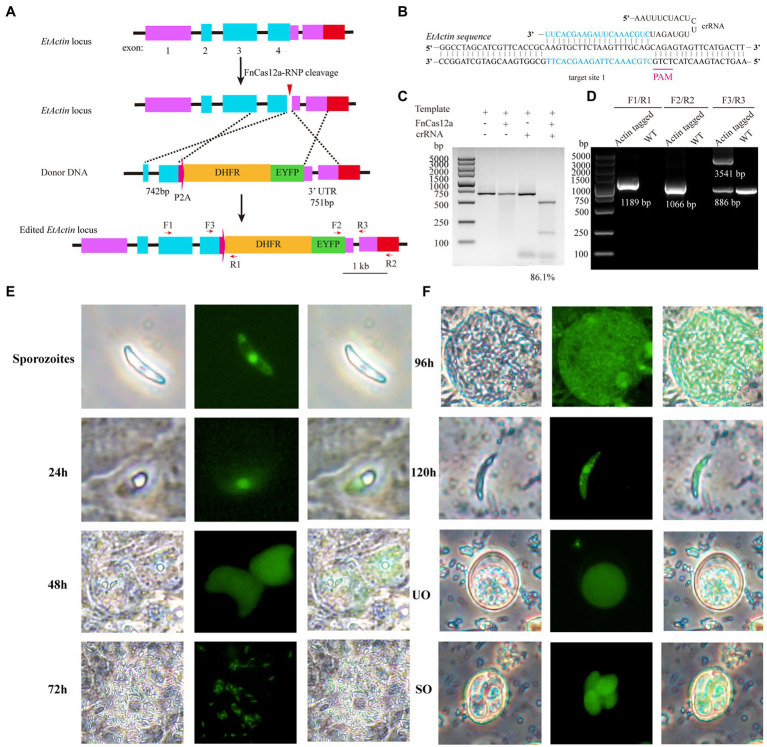
FnCas12a-mediated *EtActin* gene knock-in. **(A)** Schematic diagram illustrates FnCas12a-mediated knock-in at *EtActin*. *E. tenella* sporozoites were transfected with FnCas12a-RNP and homologous recombination template (P2A-DHFR-EYFP cassette in-frame with C-terminal of *EtActin* gene). **(B)** Schematic diagram of FnCas12a crRNA-DNA-targeting complex. The target sequence is in blue, and the PAM sequence is in pink. **(C)** *In vitro* cleavage of PCR products harboring the targeting sequence at *EtActin*. **(D)** PCR confirmation of *EtActin* tagged *E. tenella* and parental one (wild-type, WT). The positions of primers (F1/R1; F2/R2; and F3/R3) are shown in **(A)**. **(E)** Sporozoites of *EtActin* knock-in were cultured in MDBK cells and *EYFP* expression at different time points (24, 48, and 72h) after sporozoites invasion. **(F)** The cecal smears of *EtActin* knock-in were prepared in 96, 120, 144, and 168h (h.p.i.) for detection of merozoites, unsporulated oocyst, respectively. UO, unsporulated oocyst; SO, sporulated oocyst.

The above-described results showed that the endogenous gene of *E. tenella* could be targeted, although with low efficiency. To enrich the positive edited recombinants, one strategy would be using drug-mediated selection. To test it, we took advantage of the well-characterized *DHFR* gene and the drug pyrimethamine to enrich the edited products. We titrated the dose of pyrimethamine and found that *E. tenella* could be killed completely *in vivo* at the dosage of 250mg/kg pyrimethamine in feed (Supplementary Figure S4). *Actin* gene, encoding products of a central cytoskeletal component, plays an important role in several cellular processes, such as cell motility, intracellular trafficking, cytokinesis, and cell shape regulation, as target to investigate site-directed integration (knock-in; Gupta et al., 2015; Bendes et al., 2020). Donor plasmids (p*Actin*-P2A-DHFR-EYFP) were assembled, which contain the in-frame expression cassette of P2A-DHFR-EYFP with *EtActin* (Figure 3A). One crRNA to target the *EtActin* gene with a 5'-TCTG-3' PAM sequence was designed and synthesized (Figure 3B). The crRNA activity was tested *in vitro* by cleavage of the PCR fragments containing the target site (Figure 3C). The purified *E. tenella* sporozoites (1×10^7^) were transfected with 30μg of FnCas12a/crRNA (1:1) and 40μg of the linearized donor plasmids and then incubated at 41°C for 15min. The transfected sporozoites were inoculated into chickens and selected with pyrimethamine. The results of PCR analysis with the genomic DNA isolated from the transfected parasites and the parental strain (control) revealed that we have successfully knocked in the P2A-DHFR-EYFP expressing cassette with FnCas12a (Figure 3D), which was further confirmed by DNA sequencing (Supplementary Figure S5). Under the fluorescence microscopy, we found *EtActin* expressing *EYFP* in all stages of *E. tenella*, and *EYFP* was uniformly distributed in unsporulated oocysts (Figures 3E,F). The purified edited sporozoites (*Actin*-P2A-DHFR-EYFP) were cultured in MDBK cells to track *EYFP* expression after sporozoite invasion. As *E. tenella* can only be cultured to the first generation of merozoites in MDBK cells and cannot proliferate further, cecal smears were prepared at 96, 120, 144, and 168h to detect *EYFP* expression in second-generation merozoites and unsporulated merozoites. *EYFP* expression in the first- and second-generation merozoites was primarily located in the nucleus, and the expression was low in the cytoplasm (Figures 3E,F). Collectively, these results demonstrated that using FnCas12a–RNP, we can knock-in a gene to tag an endogenous gene of interest.

## Discussion

In the present study, we developed a method of FnCas12a RNP-mediated genome editing and successfully applied it to modify two genes (*EtHistone H4* and *EtActin*) of *E. tenella*. This technique may be further applied to other *Eimeria* species, which accelerates the functional genomics study and development of vaccines/drugs for *Eimeria* species, i.e., the site-specific *EYFP* knock-in strain generated here can be harnessed for high-throughput screening to identify anticoccidial drugs.

Although CRISPR/Cas as a genome editing tool was initially reported during the early 2013 (Cong et al., 2013), its application in *Eimeria* species was not achieved until last year. Of note, the relatively low transfection efficiency may contribute to this limitation. Therefore, in the present study, we optimized the conditions for transfection. The programs EO100, EH100, and EO114 in Lonza 4D-Nucleofector have higher transfection efficiency than others (Supplementary Figure S6). So far, there are two reported genome editing studies (Hu et al., 2020; Tang et al., 2020). We summarized it as Supplementary Table S3. According to the table, even with the expression of the integrated eCas9–EYFP, only 2% edited *E. tenella* are *EYFP* negative, indicating the transfection efficiency would be major bottleneck that precludes robust editing. In our study, the efficiency of *EtHistone H4* genome editing triggered with FnCas12a RNP was 1.2–1.5%. As to the knock-in at *EtActin*, gene trap strategy was used, in which only tagged recombinants would survive selection. However, due to different genome editing tools, different strategies, and different target genes, it is hard to compare genome editing efficiencies across studies. Our editing strategy is based on the RNP, which may be more beneficial for vaccines development to avoid DNA contamination.

Cas12a-mediated genome editing has been applied in *P. falciparum* to edit *EXP1*, *SirB*, and *ARP6* genes by transfection with plasmids (Nessel et al., 2020; Zhao et al., 2020). Due to PAM recognition, theoretically, there are more editable sites for AsCpf1 than SpCas9 after analyzing the genome of *P. falciparum* (Zhao et al., 2020). SpCas9 RNP has been used for the genome manipulation of *P. falciparum* (Crawford et al., 2017), and there is a report of using SaCas9 RNP to manipulate the genome of *T. cruzi*, but with SpCas9 RNP, it failed (Soares Medeiros et al., 2017). These results revealed that for a specific species, the selection of a suitable genome editing tool is an important consideration. Moreover, the results highlight that additional studies are still required for the optimization of genome editing tool for parasites.

Regarding the DSBs triggered with Cas9 or Cas12a, DNA repair mechanisms are primarily mediated by two distinct pathways (NHEJ and HDR) in mammalian cells. However, for certain parasites, e.g., *P. falciparum*, NHEJ does not exist, and hence, only HDR could be adopted for DNA repair (Zhang et al., 2014). Our *in vivo* results obtained *via* targeting *EtHistone H4* and *EtActin*, respectively, demonstrated both HDR and NHEJ are present in *E. tenella*, which provides an important insight for the genome manipulation of *Eimeria*. We acknowledge that the editing efficiency of *EtHistone H4 via* NHEJ is still low; therefore, we only obtained the sequence information *via* NGS. Of note, the EYFP-tagged *E. tenella* described in this study possessing a defined genetic background could be used for optimizing programmable editing, i.e., adenine base editors (Gaudelli et al., 2017), cytidine base editors (Komor et al., 2016), and prime editing (Anzalone et al., 2019). In particular, to select for positive genome editing events *in vivo*, a selection gene, i.e., DHFR plus pyrimethamine, may still be required.

We acknowledge the low gene editing efficiency of FnCas12a in *Eimeria*. We speculated that at least three strategies could be utilized to improve the efficiency, including using a single multiplex crRNA array for FnCas12a-mediated genome editing (Sun et al., 2018); inactivation of NHEJ pathway, i.e., disruption of KU80 (Fox et al., 2009); small molecules enhancing CRISPR-mediated editing (Yu et al., 2015).

We observed that FnCas12a exerts activity in the temperature range of 16–80°C. It also possesses much higher activity at 41–50°C than at other temperatures. This feature has a significant implication for *in vivo* genome editing studies, as the body temperature of chickens is 42°C. We speculated that FnCas12a may be more suitable than SpCas9 for *in vivo* genome editing to generate chicken disease models or gene therapy to treat chicken disease models.

In conclusion, we reported FnCas12a-RNP-mediated genome editing in *E. tenella* for the first time. Our platform would pave the way for the delineation of the gene function of *Eimeria* species, which would accelerate the development of anticoccidial drugs and vaccines.

## Data Availability Statement

The datasets presented in this study can be found in online repositories. The names of the repository/repositories and accession number(s) can be found at: https://www.ncbi.nlm.nih.gov/, SAMN19296119 and SAMN19296120.

## Ethics Statement

The animal study was reviewed and approved by Ethics Committee of Shanghai Veterinary Research Institute.

## Author Contributions

PC and FG conceived the idea and wrote the manuscript. PC, ZZ, SC, LW, and CW performed the experiments. PC, FY, MW, YL, CF, LZ, FX, and FG performed the data analyses. All authors contributed to the article and approved the submitted version.

## Funding

This research was supported by the National Natural Science Foundation of China (31871247 and 32071443), Public-Interest Scientific Institution Basal Research Fund (Y2019PT10, 2019JB04, and Y2020XK18), and Chinese Academy of Agricultural Sciences grants (CAAS-Y2019YJ07-03).

## Conflict of Interest

The authors declare that the research was conducted in the absence of any commercial or financial relationships that could be construed as a potential conflict of interest.

## Publisher’s Note

All claims expressed in this article are solely those of the authors and do not necessarily represent those of their affiliated organizations, or those of the publisher, the editors and the reviewers. Any product that may be evaluated in this article, or claim that may be made by its manufacturer, is not guaranteed or endorsed by the publisher.

## Footnotes

^1^ http://www.rgenome.net/cas-offinder/

## Abbreviations

CRISPR/Cas: Clustered regularly interspaced short palindromic repeats (CRISPR)/associated endonuclease (Cas)
DSB: Double-strand break
sgRNA: Single-guide RNA
NHEJ: Nonhomologous end-joining pathway
HDR: Homology-directed repair
*DHFR*: Dihydrofolate reductase–thymidylate
SpCas9: Streptococcus pyogenes Cas9
PAM: Protospacer adjacent motif
DR: Direct repeat
FnCas12a-RNP: FnCas12a protein and crRNA
*EYFP*: Enhanced yellow fluorescent protein
PCK: Primary chicken kidney cells
MDBK: Madin–Darby bovine kidney cells
SRA: Sequence Read Archive
*EtHistone H4*: Histone H4 gene of *E. tenella*
*EtActin*: Actin gene of *E. tenella*
*E. tenella*: *Eimeria tenella*
*P. falciparum*: *Plasmodium falciparum*
*T. cruzi*: *Trypanosoma cruzi*
