# Supplementary material for: FnCas12a/crRNA-Mediated Genome Editing in Eimeria tenella
Source: Front Genet. 2021 Sep 22;12:738746. doi: 10.3389/fgene.2021.738746 (PMC8494306; doi:10.3389/fgene.2021.738746)
Supplement: Supplementary file 1 [file Data_Sheet_1.DOCX]

Supplementary Material

**Supplementary Figures and Tables**

Figure S1.

Figure S2.

Figure S3.

Figure S4.

Figure S5.

Figure S6.

Table S1.

Table S2.

Table S3.

Supplementary protocol

The plasmid sequence for FnCas12a expression in *E coli*.

**
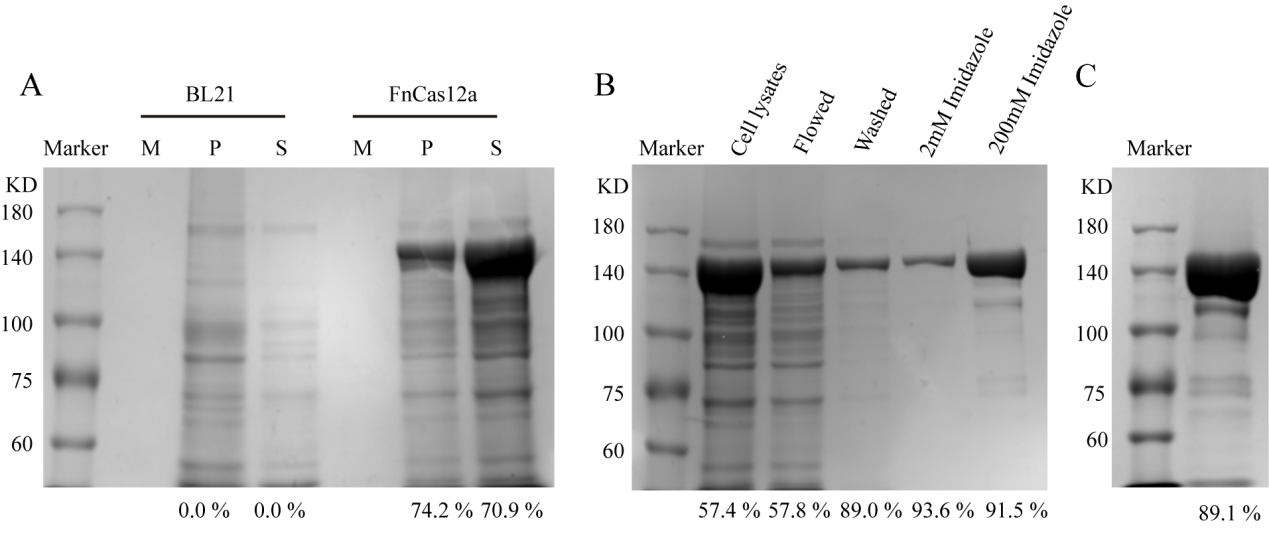
**

Figure S1: Expression and purification of FnCas12a. A, Expression of FnCas12a in *E.coli*. M, P, S represent culture medium for *E.coli*, the precipitates of cell lysates, the supernatant of cell lysates, respectively. BL21 is the host cells of *E.coli* for the ectopic expression as negative control. FnCas12a represents *E.coli* BL21 harboring the FnCas12a expression plasmid. B, FnCas12a expression in each step of purification. C, The purified FnCas12a protein. Purity of each lane was provided.


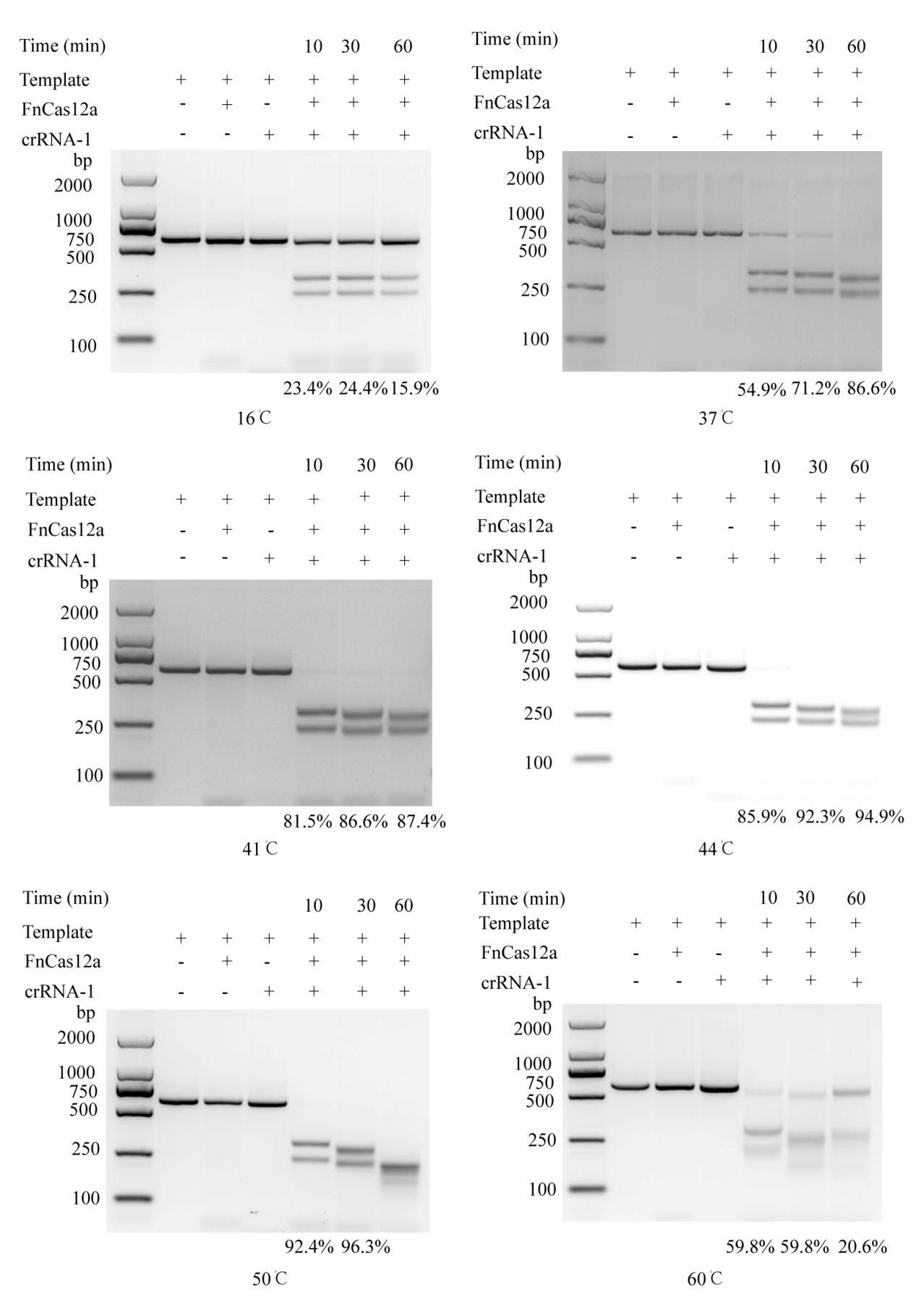


Figure S2: The effects of reaction temperature for the cleavage. The cleavage efficiencies were provided at the bottom of each lane.


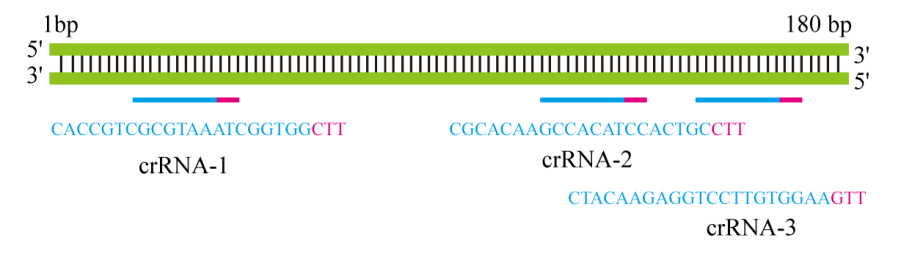


Figure S3: The target sequence of crRNA-1, crRNA-2 and crRNA-3 in *EtHistone H4*. Target and PAM sequences are in blue and purple, respectively. Here only partial DNA sequence of *EtHistone H4* is showed.


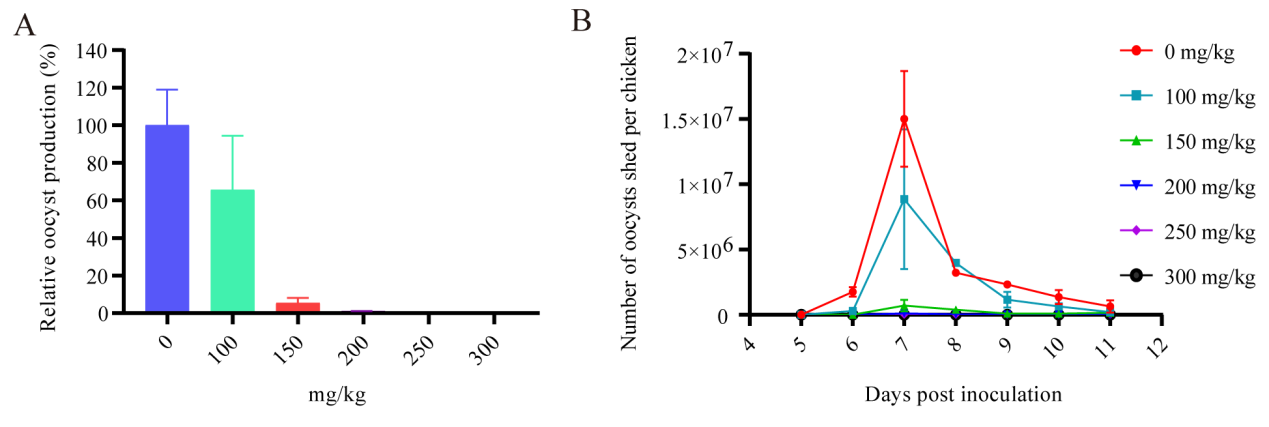


Figure S4: The optimization of pyrimethamine dosage for selection*.* A, The relative oocyst production (ROP) with pyrimethamine treatment at different dosage. The results presented as mean ± SEM from three biological replicates. B, The oocyst output curves of pyrimethamine treatment at different dosage. The oocyst outputs were presented as mean ± SEM in triplicates.


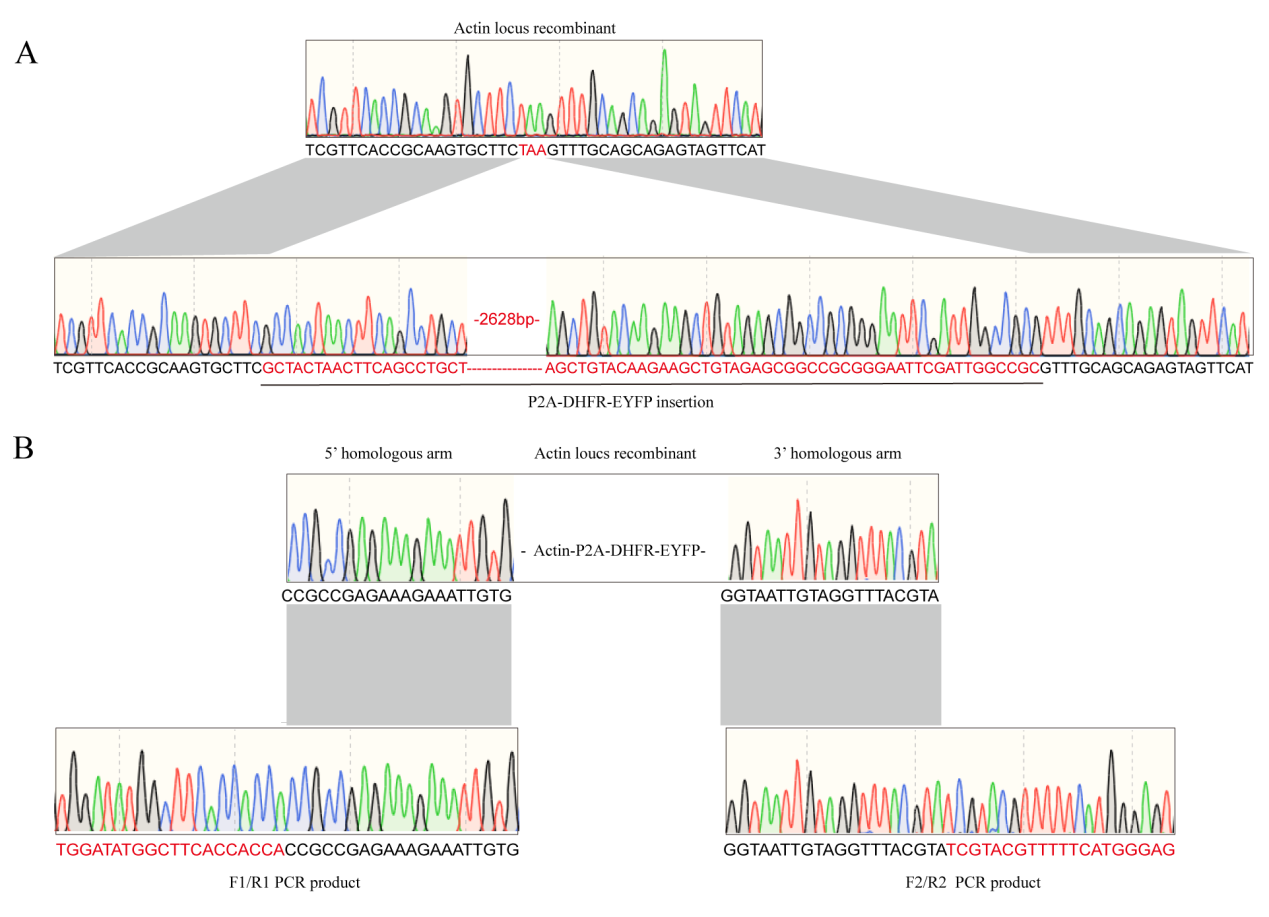


Figure S5: The results of DNA sequencing for *EtActin* knock-in. A, Confirmation of DNA sequencing. The top panel shows the nucleotide sequence between the left and right arms from parental strain (WT), and the bottom panel shows the P2A-DHFR-EYFP expression cassette knock-in (shown in red) at the C-terminal of *EtActin* gene. B, DNA sequencing confirmation of homologous recombination at *EtActin* gene edited parasites. The top panel shows the partial nucleotide sequence of the left and right arms from the homologous recombination template. The bottom panel shows the integration of homologous recombination template *Actin*-P2A-DHFR-EYFP in *EtActin* gene edited parasites. The red sequence is the genomic region of *EtActin* gene linked left and right arms of homologous recombination template.


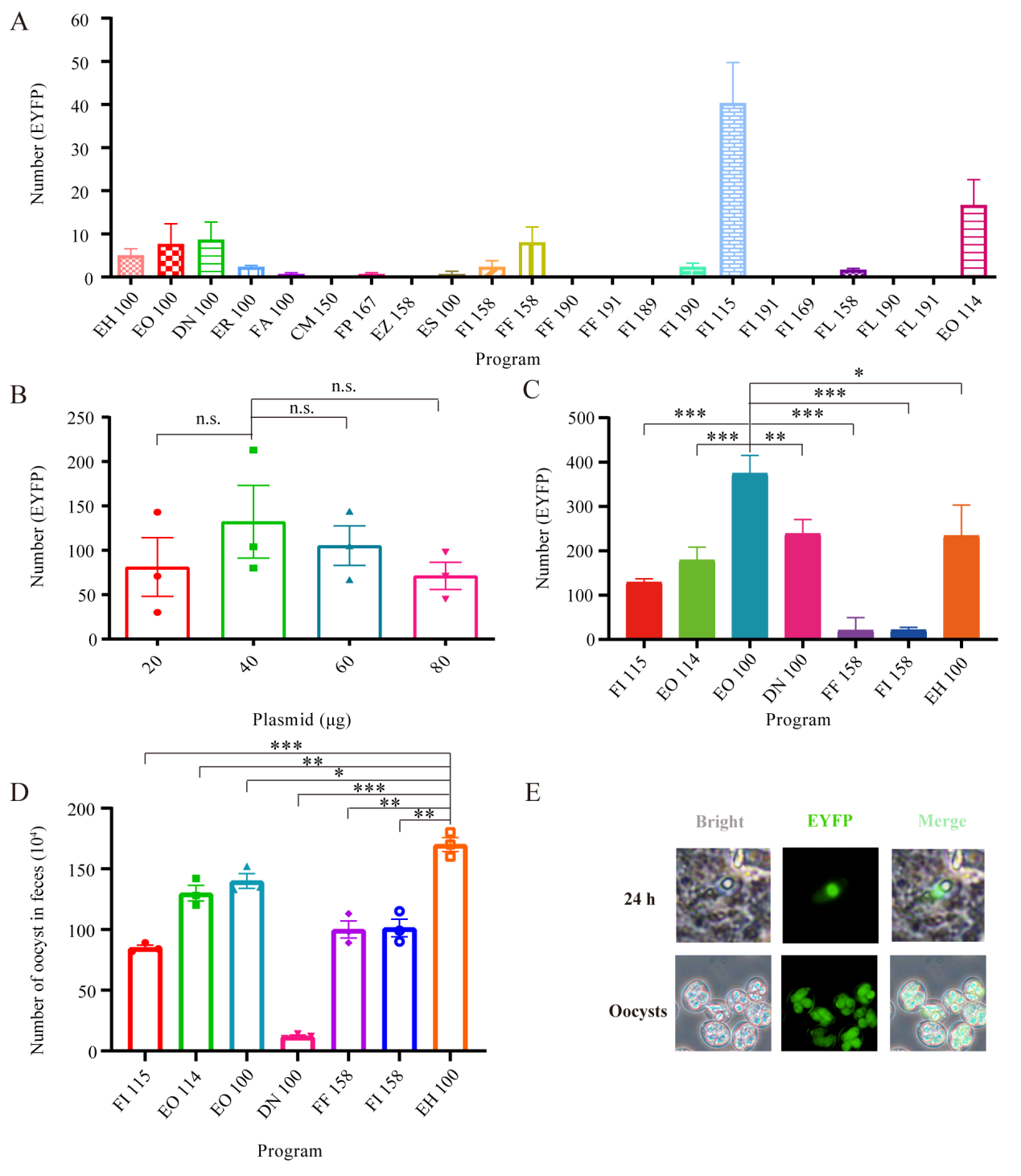


Figure S6: The optimization of transfection programs in Lonza 4D nucleofector.

A, The transfection efficiency of 22 programs were assessed in *E.tenella* sporozoites using 16-well Nucleocuvette^TM^ Strips with P3 buffer (2×10^6^ *E.tenella* sporozoites, 2 µg linearized plasmid, 1 µL SnaBI restriction enzyme). The transfection sporozoites (2×10^6^) were cultured in PCK cells and counted the number of *EYFP* expression sporozoites in 10 field of view (20×) by three times. The experiments were performed three times. The results were showed as mean ± SEM. B, The effects of plasmid amount with the program of FI 115 (1×10^7^ *E.tenella* sporozoites, 20 µg; 40 µg; 60 µg; 80 µg linearized plasmid, 5 µL SnaBI restriction enzyme). C, The comparison of transfection efficiency with seven programs *in vitro* (40 µg linearized plasmid). D, The oocysts with *EYFP* expression of the second passage. The results were showed as mean ± SEM in triplicates. E, Sporozoites of 24 h cultured in PCK cells after transfection and oocysts with *EYFP* expression at third passage after transfection. Error bars: SEM; n = 3. *P < 0.05, **P < 0.01, ***P < 0.001.

Tables

Table S1: Primers used in this study.

| Forward (F)  /Reverse (R) | Primer sequence (5'-3') | PCR product (bp) | Description |
| --- | --- | --- | --- |
| F1 | TGTGTCCCACACTGTTCCTA | 1189 (F1/R1) | *EtActin* tracing 5' integration |
| R1 | TGCTTTCCCAGTTTTTCCGT |  |  |
| F2 | GAACGGCATCAAGGTGAACT | 1066 (F2/R2) | *EtActin* tracing 3' integration |
| R2 | CCACAACTTTCCGTCACACT |  |  |
| F3 | CCGGTTCTAACTGCGTCTTT | 3541 (F3/R3) | *EtActin* tracing p2A-DHFR-EYFP inserted |
| R3 | TCTCTGGGTTCACAATGCAC |  |  |
| F4 | AAGCTTAAGCAAGACTACAGTGAACGCG | 1037 (F4/R4) | *EtHistone H*4 promoter |
| R4 | ATGGTACCTTTGGTTTTCTATGGAACAGACACAACAGACAG |  |  |
| F5 | AAAGCTTCCGCCGAGAAAGAAATTGTGC | 783 (F5/R5) | *EtActin* homology left arm |
| R5 | CTCCAGCCTGCTTCAGCAGGCTGAAGTTAGTAGCGAAGCACTTGCGGTGAACGATGCTC |  |  |
| F6 | GGAGGAGAACCCTGGACCTCAGAAGCCGGTGTGTCTGGTCGTCGC | 2602 (F6/R6) | DHER-EYFP cassette |
| R6 | CCCGCGGCCGCTCTACAGCTTCTTGTACAGCTC |  |  |
| F7 | GCTACTAACTTCAGCCTGCTGAAGCAGGCTGGAGACGTGGAGGAGAACCCTGGACCT | 2640 (F7/R6) | P2A-DHFR-EYFP  cassette |
| F8 | GTAGAGCGGCCGCGGGAATTCGATTGGCCGCGTTTG | 782 (F8/R8) | 3' *Actin* |
| R8 | TACGTAAACCTACAATTACCTGTTGCTTCGCAATC |  |  |
| F9 | CGACAACATCCAGGGTATCAC | 436 (F9/R9) | *EtHistone* *H4* PCR fragment for in vitro DNA cleavage |
| R9 | CAGCCCAACAGGCAATTTAAC |  |  |
| R10 | ACAACGGTTCCTGATGAGGTGGTT | 575 (F9/R10) | *EtHistone* *H4* PCR fragment amplification from vector pJET1.2 for in vitro DNA cleavage |
| F11 | GTCACCGCTATGGACATCGT | 153 (F11/R11) | The primers for *EtHistone* *H4* edited target deep sequencing |
| R11 | AGCTAGTTTCGCATTCGTTATTCG |  |  |

Table S2: The crRNA sequences of *EtHistone H4* and *EtActin* gene.

| Primer Description | Primer sequence (5'-3') |
| --- | --- |
| *EtHistone H4* crRNA-1 | ggtggctaaatgcgctgccac |
| *EtHistone* *H4* crRNA-2 | cgtcacctacaccgaacacgc |
| *EtHistone* *H4* crRNA-3 | aaggtgttcctggagaacatc |
| *EtActin* crRNA-1 | ctgcaaacttagaagcactt |

Table S3. The comparison of reported genome editing methods in *E.tenella.*

| Literature | Repair methods | Edited gene | Efficiency in transient experiments | Efficiency after selecting with pyrimethamine |
| --- | --- | --- | --- | --- |
| CRISPR/eCas9  (Hu et al., 2020) | NHEJ | *EYFP* | 2% | - |
|  | HDR | *EYFP* | 4% | 72% （three generations） |
|  | HDR | *EtGRA9* | - | - |
| CRISPR/SpCas9  (Tang et al., 2020) | NHEJ | *EYFP* | 1.2×10^-3^ | reduced to 89.5% （four generations） |
|  | HDR | *EtMic2* | 2.1×10^-4^ | 25.8% （three generations） |
| FnCas12a  (in the present study) | NHEJ | *EtHistone H4* | 1.2% | - |
|  | HDR  (gene trap) | *EtActin* | - | 100% （three generations） |

Supplementary protocol:

1. The purification protocol of FnCas12a protein

A single colony of recombinant *E.coli* BL21 harboring the FnCas12a expression plasmid was picked and inoculated into Luria-Bertani medium with chloramphenicol in the proportion of 1:100. At an optical density of 0.6-1.0 at 600 nm (OD_600_), IPTG 0.1 mM was added in the medium, and BL21 was cultured at 25 °C for 24 h. The cells were harvested, washed, and resuspended in nondenaturing pellets buffer. The cell pellets were lysed by sonication (200 w, on 5 s; off 5 s) in ice bath and centrifuged at 12000 rpm for 20 min at 4 °C. Then, the supernatants of FnCas12a was mixed with BeyoGold His-tag Purification Resin in 1 mM DTT 4 h at 4 °C, then washed in 10×volume of lysis buffer (300 mM NaCl, 50mM NaH_2_PO_4_, PH 8.0), 20×volume of wash buffer (300 mM NaCl, 50 mM NaH_2_PO_4_, 2 mM imidazole, PH 8.0), and eluted with 200 mM imidazole elution buffer (200 mM imidazole, 50mM NaH_2_PO_4_, 300 mM NaCl, PH 8.0). The elution sample was dialyzed with PBS without imidazole for 48 h and concentrated by PEG 20000. The concentration of FnCas12a was measured by BCA Protein Assay Kit (Beyotime Biotechnology, China). The recombinant FnCas12a proteins were analyzed by sodium dodecyl sulfate-polyacrylamide gel electrophoresis (SDS-PAGE) and coomassie blue staining.

2. Optimization of transfection programs

Initially, 22 transfection programs (EH100, EO100, DN100, ER100, FA100, CM150, FP167, EZ158, ES100, FI158, FF158, FF190, FF191, FI189, FI190, FI115, FI191, FI169, FL158, FL190, FL191, EO114) were assessed using the Lonza 4D-Nucleofector with small transfection volume (20 µL). The transfection was performed using 16-well Nucleocuvette^TM^ Strips with P3 buffer plus 2×10^6^ *E.tenella* sporozoites, 2 µg linearized plasmids (carrying *EtHistone* *H4* promoter and DHFR-EYFP expression cassettes), 1 µL SnaBI restriction enzyme. The transfected sporozoites (2×10^6^) were cultured in PCK cells and manually counted the number of *EYFP* expression sporozoites in 10 field of view (20×) by three times at 24 h post infection. Then, we used the program FI 115 to optimize the amount of plasmid (1×10^7^ *E.tenella* sporozoites, 5 µL SnaBI restriction enzyme, 20 µg; 40 µg; 60 µg; 80 µg linearized plasmid, respectively). The transfected parasites (2×10^6^) were cultured in Primary chicken kidney (PCK) cells which were prepared in six-well plate to observe the transfection efficiency. Next, we further optimized the programs (FI115, EO114, EO100, DN100, FF158, FI158, EH100) using 100 µL transfection system (1×10^7^ *Eimeria tenella* sporozoites). The EYFP expression sporozoites were counted as the above method to compare the transfection efficiency *in vitro*. Then, the rest of transfected sporozoites (8×10^6^) were inoculated to chicken and selected by Pyrimethamine. The *EYFP* expression oocysts were harvested and counted.

We found FI115, EO114, EO100, DN100, FF158, FI158, EH100 have higher transfection efficiency *in vitro*. Then these seven were further tested with augment transfection system (100 µL) and compared the transfection efficiency i*n vivo and in vitro* (Figure S6). These results suggested that EO100, EH100 and EO114 have the higher transfection efficiency.

The plasmid sequence for FnCas12a expression in *E coli*.

ggggaattgtgagcggataacaattcccctgtagaaataattttgtttaactttaataaggagatataccatgagcatctaccaggagttcgtcaacaagtattcactgagtaagacactgcggttcgagctgatcccacagggcaagacactggagaacatcaaggcccgaggcctgattctggacgatgagaagcgggcaaaagactataagaaagccaagcagatcattgataaataccaccagttctttatcgaggaaattctgagctccgtgtgcatcagtgaggatctgctgcagaattactcagacgtgtacttcaagctgaagaagagcgacgatgacaacctgcagaaggacttcaagtccgccaaggacaccatcaagaaacagattagcgagtacatcaaggactccgaaaagtttaaaaatctgttcaaccagaatctgatcgatgctaagaaaggccaggagtccgacctgatcctgtggctgaaacagtctaaggacaatgggattgaactgttcaaggctaactccgatatcactgatattgacgaggcactggaaatcatcaagagcttcaagggatggaccacatactttaaaggcttccacgagaaccgcaagaacgtgtactccagcaacgacattcctacctccatcatctaccgaatcgtcgatgacaatctgccaaagttcctggagaacaaggccaaatatgaatctctgaaggacaaagctcccgaggcaattaattacgaacagatcaagaaagatctggctgaggaactgacattcgatatcgactataagactagcgaggtgaaccagagggtcttttccctggacgaggtgtttgaaatcgccaatttcaacaattacctgaaccagtccggcattactaaattcaataccatcattggcgggaagtttgtgaacggggagaataccaagcgcaagggaattaacgaatacatcaatctgtatagccagcagatcaacgacaaaactctgaagaaatacaagatgtctgtgctgttcaaacagatcctgagtgataccgagtccaagtcttttgtcattgataaactggaagatgactcagacgtggtcactaccatgcagagcttttatgagcagatcgccgctttcaagacagtggaggaaaaatctattaaggaaactctgagtctgctgttcgatgacctgaaagcccagaagctggacctgagtaagatctacttcaaaaacgataagagtctgacagacctgtcacagcaggtgtttgatgactattccgtgattgggaccgccgtcctggagtacattacacagcagatcgctccaaagaacctggataatccctctaagaaagagcaggaactgatcgctaagaaaaccgagaaggcaaaatatctgagtctggaaacaattaagctggcactggaggagttcaacaagcacagggatattgacaaacagtgccgctttgaggaaatcctggccaacttcgcagccatccccatgatttttgatgagatcgcccagaacaaagacaatctggctcagatcagtattaagtaccagaaccagggcaagaaagacctgctgcaggcttcagcagaagatgacgtgaaagccatcaaggatctgctggaccagaccaacaatctgctgcacaagctgaaaatcttccatattagtcagtcagaggataaggctaatatcctggataaagacgaacacttctacctggtgttcgaggaatgttacttcgagctggcaaacattgtccccctgtataacaagattaggaactacatcacacagaagccttactctgacgagaagtttaaactgaacttcgaaaatagtaccctggccaacgggtgggataagaacaaggagcctgacaacacagctatcctgttcatcaaggatgacaagtactatctgggagtgatgaataagaaaaacaataagatcttcgatgacaaagccattaaggagaacaaaggggaaggatacaagaaaatcgtgtataagctgctgcccggcgcaaataagatgctgcctaaggtgttcttcagcgccaagagtatcaaattctacaacccatccgaggacatcctgcggattagaaatcactcaacacatactaagaacgggagcccccagaagggatatgagaaatttgagttcaacatcgaggattgcaggaagtttattgacttctacaagcagagcatctccaaacaccctgaatggaaggattttggcttccggttttccgacacacagagatataactctatcgacgagttctaccgcgaggtggaaaatcaggggtataagctgacttttgagaacatttctgaaagttacatcgacagcgtggtcaatcagggaaagctgtacctgttccagatctataacaaagatttttcagcatacagcaagggcagaccaaacctgcatacactgtactggaaggccctgttcgatgagaggaatctgcaggacgtggtctataaactgaacggagaggccgaactgttttaccggaagcagtctattcctaagaaaatcactcacccagctaaggaggccatcgctaacaagaacaaggacaatcctaagaaagagagcgtgttcgaatacgatctgattaaggacaagcggttcaccgaagataagttctttttccattgtccaatcaccattaacttcaagtcaagcggcgctaacaagttcaacgacgagatcaatctgctgctgaaggaaaaagcaaacgatgtgcacatcctgagcattgaccgaggagagcggcatctggcctactataccctggtggatggcaaagggaatatcattaagcaggatacattcaacatcattggcaatgaccggatgaaaaccaactaccacgataaactggctgcaatcgagaaggatagagactcagctaggaaggactggaagaaaatcaacaacattaaggagatgaaggaaggctatctgagccaggtggtccatgagattgcaaagctggtcatcgaatacaatgccattgtggtgttcgaggatctgaacttcggctttaagagggggcgctttaaggtggaaaaacaggtctatcagaagctggagaaaatgctgatcgaaaagctgaattacctggtgtttaaagataacgagttcgacaagaccggaggcgtcctgagagcctaccagctgacagctccctttgaaactttcaagaaaatgggaaaacagacaggcatcatctactatgtgccagccggattcacttccaagatctgccccgtgaccggctttgtcaaccagctgtaccctaaatatgagtcagtgagcaagtcccaggaatttttcagcaagttcgataagatctgttataatctggacaaggggtacttcgagttttccttcgattacaagaacttcggcgacaaggccgctaaggggaaatggaccattgcctccttcggatctcgcctgatcaactttcgaaattccgataaaaaccacaattgggacactagggaggtgtacccaaccaaggagctggaaaagctgctgaaagactactctatcgagtatggacatggcgaatgcatcaaggcagccatctgtggcgagagtgataagaaatttttcgccaagctgacctcagtgctgaatacaatcctgcagatgcggaactcaaagaccgggacagaactggactatctgattagccccgtggctgatgtcaacggaaacttcttcgacagcagacaggcacccaaaaatatgcctcaggatgcagacgccaacggggcctaccacatcgggctgaagggactgatgctgctgggccggatcaagaacaatcaggaggggaagaagctgaacctggtcattaagaacgaggaatacttcgagtttgtccagaatagaaataacaaaaggccggcggccacgaaaaaggccggccaggcaaaaaagaaaaagggatccggaCACCATCACCATCACCATtaagaattctgcagatatccagcacagtggcggccgcataatgcttaagtcgaacagaaagtaatcgtattgtacacggccgcataatcgaaattaatacgactcactatagGGaatttctactgttgtagattgagaccacggcaggtctctAATTTCTACTGTTGTAGATccgctgagcaataactagcataaccccttggggcctctaaacgggtcttgaggggttttttgctgaaacctcaggcatttgagaagcacacggtcacactgcttccggtagtcaataaaccggtaaaccagcaatagacataagcggctatttaacgaccctgccctgaaccgacgaccgggtcgaatttgctttcgaatttctgccattcatccgcttattatcacttattcaggcgtagcaaccaggcgtttaagggcaccaataactgccttaaaaaaattacgccccgccctgccactcatcgcagtactgttgtaattcattaagcattctgccgacatggaagccatcacaaacggcatgatgaacctgaatcgccagcggcatcagcaccttgtcgccttgcgtataatatttgcccatagtgaaaacgggggcgaagaagttgtccatattggccacgtttaaatcaaaactggtgaaactcacccagggattggctgagacgaaaaacatattctcaataaaccctttagggaaataggccaggttttcaccgtaacacgccacatcttgcgaatatatgtgtagaaactgccggaaatcgtcgtggtattcactccagagcgatgaaaacgtttcagtttgctcatggaaaacggtgtaacaagggtgaacactatcccatatcaccagctcaccgtctttcattgccatacggaactccggatgagcattcatcaggcgggcaagaatgtgaataaaggccggataaaacttgtgcttatttttctttacggtctttaaaaaggccgtaatatccagctgaacggtctggttataggtacattgagcaactgactgaaatgcctcaaaatgttctttacgatgccattgggatatatcaacggtggtatatccagtgatttttttctccattttagcttccttagctcctgaaaatctcgataactcaaaaaatacgcccggtagtgatcttatttcattatggtgaaagttggaacctcttacgtgccgatcaacgtctcattttcgccaaaagttggcccagggcttcccggtatcaacagggacaccaggatttatttattctgcgaagtgatcttccgtcacaggtatttattcggcgcaaagtgcgtcgggtgatgctgccaacttactgatttagtgtatgatggtgtttttgaggtgctccagtggcttctgtttctatcagctgtccctcctgttcagctactgacggggtggtgcgtaacggcaaaagcaccgccggacatcagcgctagcggagtgtatactggcttactatgttggcactgatgagggtgtcagtgaagtgcttcatgtggcaggagaaaaaaggctgcaccggtgcgtcagcagaatatgtgatacaggatatattccgcttcctcgctcactgactcgctacgctcggtcgttcgactgcggcgagcggaaatggcttacgaacggggcggagatttcctggaagatgccaggaagatacttaacagggaagtgagagggccgcggcaaagccgtttttccataggctccgcccccctgacaagcatcacgaaatctgacgctcaaatcagtggtggcgaaacccgacaggactataaagataccaggcgtttcccctggcggctccctcgtgcgctctcctgttcctgcctttcggtttaccggtgtcattccgctgttatggccgcgtttgtctcattccacgcctgacactcagttccgggtaggcagttcgctccaagctggactgtatgcacgaaccccccgttcagtccgaccgctgcgccttatccggtaactatcgtcttgagtccaacccggaaagacatgcaaaagcaccactggcagcagccactggtaattgatttagaggagttagtcttgaagtcatgcgccggttaaggctaaactgaaaggacaagttttggtgactgcgctcctccaagccagttacctcggttcaaagagttggtagctcagagaaccttcgaaaaaccgccctgcaaggcggttttttcgttttcagagcaagagattacgcgcagaccaaaacgatctcaagaagatcatcttattaatcagataaaatatttctagatttcagtgcaatttatctcttcaaatgtagcacctgaagtcagccccatacgatataagttgtaattctcatgttagtcatgccccgcgcccaccggaaggagctgactgggttgaaggctctcaagggcatcggtcgagatcccggtgcctaatgagtgagctaacttacattaattgcgttgcgctcactgcccgctttccagtcgggaaacctgtcgtgccagctgcattaatgaatcggccaacgcgcggggagaggcggtttgcgtattgggcgccagggtggtttttcttttcaccagtgagacgggcaacagctgattgcccttcaccgcctggccctgagagagttgcagcaagcggtccacgctggtttgccccagcaggcgaaaatcctgtttgatggtggttaacggcgggatataacatgagctgtcttcggtatcgtcgtatcccactaccgagatgtccgcaccaacgcgcagcccggactcggtaatggcgcgcattgcgcccagcgccatctgatcgttggcaaccagcatcgcagtgggaacgatgccctcattcagcatttgcatggtttgttgaaaaccggacatggcactccagtcgccttcccgttccgctatcggctgaatttgattgcgagtgagatatttatgccagccagccagacgcagacgcgccgagacagaacttaatgggcccgctaacagcgcgatttgctggtgacccaatgcgaccagatgctccacgcccagtcgcgtaccgtcttcatgggagaaaataatactgttgatgggtgtctggtcagagacatcaagaaataacgccggaacattagtgcaggcagcttccacagcaatggcatcctggtcatccagcggatagttaatgatcagcccactgacgcgttgcgcgagaagattgtgcaccgccgctttacaggcttcgacgccgcttcgttctaccatcgacaccaccacgctggcacccagttgatcggcgcgagatttaatcgccgcgacaatttgcgacggcgcgtgcagggccagactggaggtggcaacgccaatcagcaacgactgtttgcccgccagttgttgtgccacgcggttgggaatgtaattcagctccgccatcgccgcttccactttttcccgcgttttcgcagaaacgtggctggcctggttcaccacgcgggaaacggtctgataagagacaccggcatactctgcgacatcgtataacgttactggtttcacattcaccaccctgaattgactctcttccgggcgctatcatgccataccgcgaaaggttttgcgccattcgatggtgtccgggatctcgacgctctcccttatgcgactcctgcattaggaaattaatacgactcactata
